# E3 Ubiquitin Ligase Smurf1 Regulates the Inflammatory Response in Macrophages and Attenuates Hepatic Damage during Betacoronavirus Infection

**DOI:** 10.3390/pathogens13100871

**Published:** 2024-10-03

**Authors:** Luiz P. Souza-Costa, Felipe R. S. Santos, Jordane C. Pimenta, Celso M. Queiroz-Junior, Fernanda L. Tana, Danielle C. Teixeira, Manoela G. G. Couto, Natalia F. M. Oliveira, Rafaela D. Pereira, Vinicius A. Beltrami, Pedro A. C. Costa, Larisse S. B. Lacerda, Josiane T. Andrade-Chaves, Pedro P. G. Guimarães, Renato S. Aguiar, Mauro M. Teixeira, Vivian V. Costa, Luis H. Franco

**Affiliations:** 1 Departamento de Bioquímica e Imunologia, Instituto de Ciências Biológicas, Universidade Federal de Minas Gerais, Av. Pres. Antônio Carlos, 6627, Belo Horizonte 31270-901, MG, Brazil; souzaluizpedro@gmail.com (L.P.S.-C.);; 2 Departamento de Morfologia, Instituto de Ciências Biológicas, Universidade Federal de Minas Gerais, Av. Pres. Antônio Carlos, 6627, Belo Horizonte 31270-901, MG, Brazil; 3 Departamento de Fisiologia e Biofísica, Instituto de Ciências Biológicas, Universidade Federal de Minas Gerais, Av. Pres. Antônio Carlos, 6627, Belo Horizonte 31270-901, MG, Brazil; 4 Departamento de Genética e Evolução, Instituto de Ciências Biológicas, Universidade Federal de Minas Gerais, Av. Pres. Antônio Carlos, 6627, Belo Horizonte 31270-901, MG, Brazil

**Keywords:** Smurf1, Betacoronavirus, MHV-A59, inflammation, macrophages

## Abstract

The E3 ubiquitin ligase Smurf1 catalyzes the ubiquitination and proteasomal degradation of several protein substrates related to inflammatory responses and antiviral signaling. This study investigated the role of Smurf1 in modulating inflammation induced by Betacoronavirus infection. Bone marrow-derived macrophages (BMDMs) from C57BL/6 (wild-type) or Smurf1-deficient (*Smurf1*^−/−^) mice were infected with MHV-A59 to evaluate the inflammatory response in vitro. Smurf1 was found to be required to downregulate the macrophage production of pro-inflammatory mediators, including TNF, and CXCL1; to control viral release from infected cells; and to increase cell viability. To assess the impact of Smurf 1 in vivo, we evaluated the infection of mice with MHV-A59 through the intranasal route. *Smurf1*^−/−^ mice infected with a lethal inoculum of MHV-A59 succumbed earlier to infection. Intranasal inoculation with a 10-fold lower dose of MHV-A59 resulted in hematological parameter alterations in *Smurf1*^−/−^ mice suggestive of exacerbated systemic inflammation. In the lung parenchyma, Smurf1 expression was essential to promote viral clearance, downregulating IFN-β mRNA and controlling the inflammatory profile of macrophages and neutrophils. Conversely, Smurf1 did not affect IFN-β mRNA regulation in the liver, but it was required to increase TNF and iNOS expression in neutrophils and decrease TNF expression in macrophages. In addition, *Smurf1*^−/−^ mice exhibited augmented liver injuries, accompanied by high serum levels of alanine aminotransferase (ALT). These findings suggest that Smurf1 plays a critical role in regulating the inflammatory response in macrophages and attenuating systemic inflammation during Betacoronavirus infection.

## 1. Introduction

Betacoronavirus is a genus that belongs to the *Coronaviridae* family of viruses, and it includes enveloped viruses with single-stranded, positive-sense RNA. This genus comprises a vast number of members that cause zoonotic and human illnesses, ranging from the common cold to the highly severe acute respiratory coronavirus disease 2019 (COVID-19), responsible for the recent pandemic that led to nearly 7 million deaths [1]. Given the wide geographic distribution of Betacoronaviruses, the recurrence of zoonotic outbreaks, and the potential for new Betacoronaviruses to develop into pandemics, it is vital to enhance our understanding of how host immune factors regulate immune responses to Betacoronaviruses.

Murine hepatic coronavirus (MHV) is a Betacoronavirus that infects mice and closely resembles key aspects of human Betacoronavirus infection [2], being often used as a model to study immune responses against coronaviruses [3,4,5]. Murine intranasal infection with the MHV-A59 strain leads to an altered neutrophil/lymphocyte ratio in the blood and causes lung injuries associated with pneumonia, hemorrhage, hyperplasia and fibrosis of the alveolar walls, which recapitulates the acute respiratory distress syndrome caused by acute respiratory syndrome coronavirus (SARS-CoV) and Middle East respiratory syndrome coronavirus (MERS-CoV) infection in humans. MHV-A59-caused lung injuries are accompanied by infiltration of macrophages and neutrophils in the alveolar walls; the upregulation of CCL2, CCL3, CCL5, IP-10, TNF, IL-6, and IL-1β; and PANoptosis, a kind of inflammatory cell death that integrates components from pyroptosis, apoptosis, and necroptosis [3,6,7,8]. Following replication in the lungs, MHV becomes systemic and infects multiple organs, including the liver. Intranasal or intraperitoneal infection with the highly hepatotropic MHV-3 leads to intense liver damage characterized by hepatitis; necrosis; an intense infiltration of macrophages and neutrophils; increased expression of IL-6, TNF, CXCL1, CCL2, and IP-10; high serum levels of alanine aminotransferase (ALT); and death, while infection with MHV-A59 induces a comparable mild and subclinical hepatitis [5,9]. Overall, these studies indicate that the lung and liver immunopathology observed in murine Betacoronavirus infection results from a dysregulated/uncontrolled inflammatory immune response, similar to that seen in human Betacoronavirus infection [10]. Thus, understanding the mechanisms that regulate inflammatory immune responses during Betacoronavirus infection may facilitate the development of strategies to treat coronavirus disease.

Ubiquitination is a posttranslational process that consists of ubiquitin binding to protein [11] or lipid [12] substrates. Ubiquitination controls multiple homeostatic processes including protein targeting to proteasomal degradation, signal transduction cascades, and innate immune responses [13,14]. Ubiquitination is orchestrated by the action of three classes of enzymes known as E1, E2, and E3 enzymes. The E3 ubiquitin ligases control the specificity of substrate ubiquitination, as these enzymes directly interact with substrates and promote the ligation of ubiquitin to them. SMAD ubiquitin regulatory factor 1 (Smurf1) is an E3 ubiquitin ligase that catalyzes the ubiquitination and proteasomal degradation of several protein substrates related to inflammatory responses and antiviral signaling (revised by [15]), including myeloid differentiation primary response 88 (MyD88) [16]; mitochondrial antiviral signaling protein (MAVS) [17]; signal transducer and activator of transcription factor 1 (STAT1) [18];, tumor necrosis factor receptor-associated factor 3 (TRAF3); TRAF6 [19]; and USP25, a deubiquitination enzyme that interacts with TRAF3 and TRAF6 [20]. mRNA for Smurf1 is highly expressed in nasopharyngeal and oropharyngeal swabs from patients positive for SARS-CoV-2 with severe respiratory symptoms, and Smurf1 directly interacts with the SARS-CoV-2 spike protein [21], which suggest that Smurf1 may work on the regulation of immune responses during Betacoronavirus infection. Because the mechanism of how Smurf1 controls immune responses against Betacoronavirus infection needs to be further explored, in this work, we aim to evaluate the role of Smurf1 in regulating immune responses using a murine model of Betacoronavirus infection and to understand the role of Smurf1 in the immunopathogenesis of Betacoronavirus diseases. Our data brings new insights into host factors that regulate inflammatory immune responses, which could aid the development of new therapeutic strategies to avoid immunopathology caused by coronavirus infection.

## 2. Materials and Methods

### 2.1. Cell Lineage and Virus

L929 fibroblasts (ATCC CCL-1) were cultured with high-glucose Dulbecco’s Modified Eagle Medium (DMEM) (Cultilab, Campinas, Brazil) supplemented with 7% fetal bovine serum (FBS) (Biowest, Nuaillé, France), 100 U/mL penicillin, and 100 μg/mL streptomycin (Thermo Fisher Scientific, Grand Island, NE, USA) under an atmosphere of 37 °C, 5% CO_2_. The MHV-A59 strain (ATCC VR-764) was propagated in L929 cells as previously published [5].

### 2.2. Animals

C57BL/6 (wild-type) and Smurf1-deficient (*Smurf1*^−/−^*)* mice [22] (with a C57BL/6 genetic background) were housed in individually open cages placed in an animal facility at 24 ± 2 °C on a 12 h light/dark cycle, receiving ad libitum access to water and food.

The experimental proceedings were made with mixed groups (male or female) of 6- to 8-week-old mice and received approval from the Ethics Committee on Animal Experimentation of the Federal University of Minas Gerais (UFMG) (processes nº 191/2020 and 186/2022).

### 2.3. Bone Marrow-Derived Macrophages and Viral Infection

Bone marrow-derived macrophages (BMDMs) were generated from the bone marrow of C57BL/6 or *Smurf1*^−/−^ mice as previously reported [23]. Briefly, bone marrow cells from the tibia and femur were collected and cultured for seven days in an L929 cell-conditioned medium. After seven days, BMDMs were seeded in 24-well culture plates in triplicate (2 × 10^5^ cells/well). For viral infection, MHV-A59 was diluted in a multiplicity of infection (MOI) of 0.01 plate forming unit (PFU)/cell in 100 µL DMEM supplemented with 2% FBS and added to the wells. Plates were incubated for 1 h at 37 °C, 5% CO_2_ to allow virus adsorption. Then, fresh media were added to the wells to a final volume of 500 µL per well, and cells were incubated for different time points as indicated in the figures. At the end of each time point, the supernatants were collected and frozen at −80 °C for subsequent quantitation of the viral titers or inflammatory mediators.

### 2.4. Quantitation of Inflammatory Mediators

The quantitation of inflammatory mediators was assessed using the Enzyme-Linked Immunosorbent Assay (ELISA) method using the mouse Duoset system (R&D Systems, Minneapolis, MN, USA) according to the manufacturer’s instructions.

### 2.5. Animal Infection and Sample Collection

Mice were divided into infected or non-infected (mock) groups. Infection was performed by inoculating 30 µL of sterile saline solution containing either 3 × 10^4^ (low-dose inoculum) or 3 × 10^5^ (high-dose inoculum) PFU of MHV-A59 per mouse via the intranasal route. Mock groups received 30 µL of sterile saline. Mice were euthanized at 2, 5, and 11 days post-infection for sample collection. Whole blood was collected from the vena cava and placed in tubes coated with EDTA for subsequent hematological parameters analysis. The liver and lungs were harvested and rinsed in cold phosphate-buffered saline (PBS) (pH 7.4). The right lobe of the lungs and a fragment of the liver were frozen at −80 °C until processing for viral titer quantitation. The left lobe of the lungs and a piece of the liver were fixed by immersion in a 4% buffered formalin solution for subsequent histopathological analysis.

### 2.6. Survival and Weight Loss Analysis

For the survival analyses, mice were infected with a high-dose inoculum of MHV-A59 as described above, and the body weight of infected mice was measured once a day until 11 days post-infection. Mice were euthanized when they lost 20% of their initial body weight. Mice that survived after 11 days post-infection were euthanized.

### 2.7. Hematological Parameters

The number of circulating white blood cells (WBC) and the frequency of granulocytes and lymphocytes in the whole blood of mice was quantified by the Celltac MEK-6500K hemocytometer (Nihon Kohden, Tokyo, Japan) according to the manufacturer’s instructions.

### 2.8. Viral Titer Quantitation

Viral titer quantitation was performed either in the supernatants of MHV-A59-infected BMDMs or in homogenates of lung and liver from infected mice as previously reported [5]. Briefly, monolayers of the L929 fibroblast were seeded in 24-well dishes (1 × 10^5^ cells/well) and incubated overnight for adherence. On the next day, 100 µL of serially diluted BMDM supernatants or tissue homogenates were added to the monolayers and gently shaken for 1 h. Samples were removed and replaced by an overlay medium (DMEM with 1.5% *w*/*v* carboxymethylcellulose [Synth, SP, Brazil] and 2% FBS), followed by incubation at 37 °C, 5% CO_2_ for 48 h. After incubation, the cells were fixed with 10% buffered formaldehyde, washed, and stained with methylene blue (Synth, SP, Brazil) 1% *w*/*v*. Viral titer was determined by counting PFUs, expressed as Log_10_ PFU/mL for BMDM supernatant or Log_10_ PFU/100 mg of tissue for tissue samples.

### 2.9. Flow Cytometry

Macrophages and neutrophils immunophenotyping were assessed using processed and enriched lung and liver tissues. Liver tissue was macerated with a 70 μm pore cell strainer (Corning, Tewksbury, MA, USA), followed by erythrocyte lysis, and enrichment of leukocytes with a Percoll gradient (Sigma-Aldrich, St. Louis, MI, USA). Lung tissues were cut into small pieces, digested with collagenase I (Gibco, Grand Island, NE, USA), and passed through a 70 μm pore cell strainer. Liver and lung isolated cells were cultured for 4 h in RPMI plus supplements in the presence of Brefeldin A (ThermoFisher). To exclude dead cells, viable cells were stained with a live/dead marker (Acqua, Invitrogen, 1:1000). For staining of the extracellular markers, the following mAbs were used: anti-CD45 (PerCP-Cy5.5, MCD4505, ThermoFisher, 1:200); anti-CD11b (Super Bright, M1/70, ThermoFisher, 1:500); anti-F4/80 (APC, BM8, ThermoFisher, 1:200); anti-Gr-1 (Biotin, RB6-8C5, Biolegend, San Diego, CA, USA, 1:500); anti-MHC Class II (I-A/I-E) (APC-eFluor 780, M5/114.15.2, ThermoFisher, 1:400); Streptavidin (Pacific Orange, ThermoFisher, 1:200). For intracellular staining, the cells were washed, fixed, permeabilized with the Cytofix/Cytoperm buffer set (eBioscience, Carlsbad, CA, USA) according to manufacturer’s instructions, and incubated with the following antibodies: anti-TNF (PE, DY410, R&D System, McKinley Place, NM, USA, 1:500); anti-iNOS (PE-eFluor610, CXNFT, ThermoFisher, 1:500); anti-IL-10 (AlexaFluor 700, JES5-16E3, ThermoFisher, 1:100). The BD LSR-FORTESSA equipment was used for acquisition, and data were analyzed using singlets with an FSC-A port versus the FSC-H gate, with the exclusion of debris and bubble evaluation. Macrophages were gated on CD45^+^CD11b^+^Gr-1^-^F4/80^+^ live cells, and neutrophils were gated on CD45^+^CD11b^+^Gr-1^+^F4/80^-^ live cells. FlowJo V10.4.11 was used for data analysis.

### 2.10. Histopathological Analysis

Lungs and liver were fixed by immersion in a 4% buffered formalin solution for 48 h and transferred to 70% ethanol. Fixed tissues were embedded in paraffin and sectioned in 5 μm thick slices, stained with hematoxylin–eosin (H&E), and examined under light microscopy by a pathologist (C.M.Q.-J) in a blinded manner. The quantitation of inflammation-mediated injury in the lungs was made using a scoring system that encompasses (i) airway inflammation (up to 4 points); (ii) vascular inflammation (up to 4 points); (iii) parenchymal inflammation (up to 5 points); and general neutrophilic infiltration (up to 5 points). A histopathological analysis of the liver took into account the presence of the inflammatory infiltrate, vessel dilation, tissue degeneration, necrosis, and hemorrhage, where the parameters were scored as follows: 0: absent (0%); 1: minimal (1–20%), 2: mild (21–40%); 3: moderate (41–60%); 4: accentuated (61–80%); 5: severe (81–100%) [24].

### 2.11. Quantitation of Serum Alanine Aminotransferase Activity

Serum alanine aminotransferase (ALT) activity was assessed using a kinetic test (Bioclin, Belo Horizonte, MG, Brazil) according to the manufacturer instructions. Briefly, 20 µL of serum diluted in PBS (1:1) was added to each well of a 96-well plate. Subsequently, 180 mL of the substrates and coenzymes was added to the wells, and the plate was incubated at 37 °C. The absorbance was read at 340 nm. The results were expressed as the mean of four readings collected in an interval of 1 min each.

### 2.12. RT-qPCR

The evaluation of mRNA levels for IFN-β and Smurf1 in the lungs and liver of the infected mice was performed by RT-qPCR using specific primers. Tissue samples from animals infected with MHV-A59 were collected and stored at −80 °C until total RNA extraction. Subsequently, the tissue was weighed (~30 mg tissue/sample), and RNA extraction was performed using the PureLink RNA Mini Kit (Invitrogen, Carlsbad, CA, USA). After extraction, RNA was quantified using the Qubit RNA High Sensitivity Assay kit (Invitrogen), and 500 ng of RNA/sample was normalized for all samples. Then, the cDNA was prepared using the High-Capacity cDNA Reverse Transcription kit (Applied Biosystems, Vilnius, Lithuania), and RT-qPCR for the target genes was performed using PrimeTime qPCR primers and SYBR Green (Gotaq qPCR Master Mix—Promega), from 2 µL of cDNA. The assay was performed using Biorad CFX Maestro (Biorad, Hercules, CA, USA), and the results were expressed as relative expression units after normalization for the constitutive HPRT gene. The sequences of the primers used were as follows: IFN-β: Primer 1: 5’-CCCCAAAATGGTTAAGGTTGC-3’; Primer 2: 5’-AACAAAGTCTGGCCTGTATCC-3’. Smurf1: Primer 1: 5’–CCAAATAGTGGTCAGTTTTACAG-3′; Primer 2: 5′–CAGTACCATCTGTATATGGGG. HPRT: Primer 1: 5′-CCCCAAAATGGTTAAGGTTGC-3′; Primer 2: 5′-AACAAAGTCTGGCCTGTATCC-3′; TNF: Primer 1: 5’-AGACCCTCACACTCAGATCA-3’; Primer 2: 5’-TCTTTGAGATCCATGCCGTTG-3’; IL-6: Primer 1: 5’-AGCCAGAGTCCTTCAGAGA-3’; Primer 2: 5’-TCCTTAGCCACTCCTTCTGT-3’.

### 2.13. Statistical Analysis

Statistical analyses were performed using GraphPad Prism software (version 8.0.1; GraphPad, San Diego, CA, USA). The two-tailed unpaired Student’s *t*-test was used for single comparisons, and an analysis of variance (ANOVA) was used for multiple comparisons, as indicated in the figure legends. The Log-rank (Mantel-Cox) test was used for survival studies. The data are presented as mean ± standard error of the mean (SEM). Differences were considered statistically significant at *p* < 0.05.

## 3. Results

### 3.1. Smurf1 Modulates the Synthesis of Pro-Inflammatory Mediators, Viral Release, and Cell Viability in Betacoronavirus-Infected Macrophages

**Figure 1:**
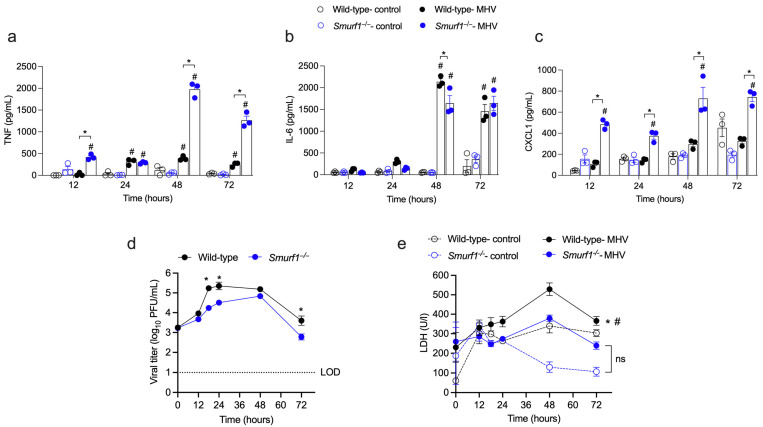
Smurf1 modulates the synthesis of pro-inflammatory mediators, virus release, and cell viability in Betacoronavirus-infected macrophages. (**a**–**c**) BMDMs from wild-type or *Smurf1*^−/−^ mice were infected with 0.01 PFU of MHV-A59 and TNF (**a**), IL-6 (**b**), and CXCL1 (**c**) levels were measured in the supernatants by ELISA. Bars represent the mean ± SEM of triplicate samples. Statistical analysis was performed using a two-way ANOVA test. # *p* < 0.05 in comparison to the respective uninfected control group; * *p* < 0.05. (**d**,**e**) BMDMs were infected as indicated above and viral titer (**d**) and LDH production (**e**) were measured in the supernatants. Data are shown as mean ± SEM of triplicate samples. Statistical analysis was performed using a two-way ANOVA test. * *p* < 0.05 when comparing data from infected wild-type and *Smurf1*^−/−^ groups; # *p* < 0.05 in comparison to the respective uninfected control group; LOD: limit of detection; ns: non-significant.

It has been shown that lung and liver immunopathology during experimental Betacoronavirus infection is accompanied by an intense infiltration of macrophages and upregulation of pro-inflammatory mediators [6,7], supporting the concept that macrophages play a critical role in pathogenesis caused by Betacoronavirus [25]. To investigate the role of Smurf1 in modulating immune responses in macrophages during Betacoronavirus infection, we infected bone marrow-derived macrophages (BMDMs) from C57BL/6 (wild-type) or *Smurf1*^−/−^ mice with MHV-A59 and quantified the production of pro-inflammatory mediators in cell culture supernatants by ELISA. As previously shown [26], MHV-A59 induced the production of TNF in wild-type BMDMs starting from 24 h post-infection. Interestingly, MHV-A59 stimulated TNF production in *Smurf1*^−/−^ BMDMs as early as 12 h post-infection, and the levels of TNF produced by *Smurf1*^−/−^ BMDMs at 48 and 72 h post-infection were five times higher than those in MHV-A59-infected wild-type BMDMs (Figure 1a). In addition, MHV-A59 stimulated the production of IL-6 by wild-type BMDMs starting from 48 h post-infection, when the levels detected by infected cells were higher than those in uninfected controls (Figure 1b). *Smurf1*^−/−^ BMDMs secreted similar levels of IL-6 compared to MHV-A59-infected wild-type in all periods evaluated, with the exception of 48 h post-infection, when IL-6 secretion by *Smurf1*^−/−^ BMDMs was lower, compared to wild-type infected cells (Figure 1b). MHV-A59 did not induce the production of CXCL1, a chemokine associated with neutrophil recruitment, in wild-type BMDMs. However, MHV-A59 stimulated the production of CXCL1 in *Smurf1*^−/−^ BMDMs as early as 12 h post-infection compared to uninfected *Smurf1*^−/−^ or MHV-A59-infected wild-type BMDMs (Figure 1c). To evaluate the impact of Smurf1 on viral release in infected macrophages, we measured viral titers in the supernatants from MHV-A59-infected BMDMs. Our data showed that the absence of Smurf1 did not interfere with viral cell attachment and entry, as the viral title was similar between wild-type and *Smurf1*^−/−^ BMDMs at time zero of infection. However, we found that supernatants from *Smurf1*^−/−^ BMDMs presented significantly lower numbers of infective viral particles starting from 18 h post-infection, compared to wild-type cells (Figure 1d). Additionally, we assessed cell viability by measuring lactate dehydrogenase (LDH) release in the supernatants of infected cells. Our findings indicate that infection with MHV-A59 induced significant levels of cell death in wild-type BMDMs, compared to uninfected cells, whereas it did not induce significant cell death in *Smurf1*^−/−^ BMDMs (Figure 1e). This effect of Smurf1 on cell death was dependent on MHV-A59 infection, as both wild-type and *Smurf1*^−/−^ uninfected cells exhibited similar levels of LDH release. Upon a comparison of LDH release in both phenotypes following infection, we observed lower levels of LDH release in cultures of *Smurf1*^−/−^ BMDMs infected with MHV-A59 (Figure 1e), indicating that *Smurf1*^−/−^ BMDMs demonstrated increased cell viability compared to wild-type macrophages upon viral infection. Overall, these data indicate that Smurf1 is involved in modulating the secretion of pro-inflammatory mediators, facilitating viral release, and increasing cell viability in macrophages during Betacoronavirus infection.

### 3.2. Smurf1 Is Required for Host Resistance to Betacoronavirus Infection and Regulation of Systemic Inflammation

**Figure 2:**
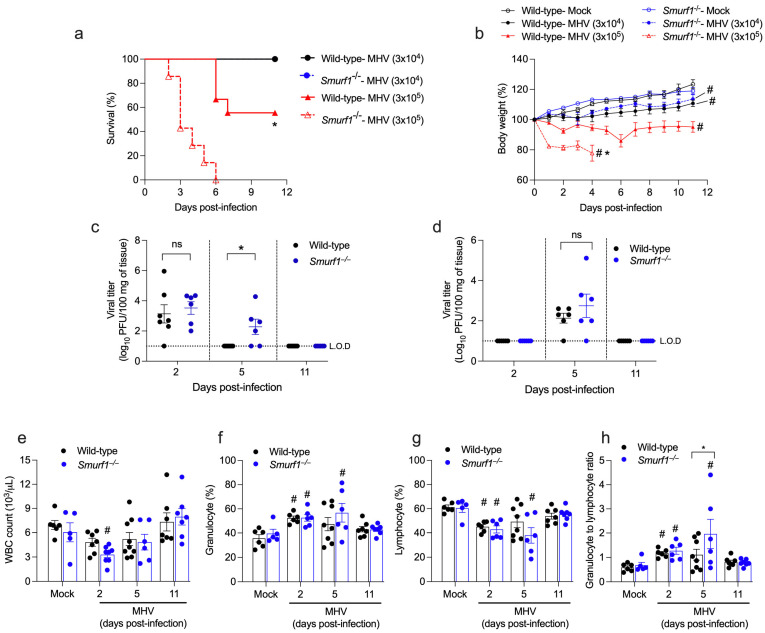
Smurf1 is required for host resistance to Betacoronavirus infection and regulation of systemic inflammation. (**a**) Survival curve from mice inoculated via the intranasal route with 3 × 10^4^ (low-dose inoculum) or 3 × 10^5^ (high-dose inoculum) PFU of MHV-A59. * *p* < 0.05 when compared to the *Smurf1*^−/−^ group infected with 3 × 10^5^ PFU of MHV-A59. Log-rank test; n = 7–9 mice/group. (**b**) Body weight loss in MHV-A59-infected mice. Data represent mean ± SEM; n = 5–9 mice/group. #*p* < 0.05 when compared to their respective mock group; * *p* < 0.05 when compared to wild-type mice infected with 3 × 10^5^ PFU of MHV-A59. Two-way ANOVA test. (**c**,**d**) Viral titers in the lungs (**c**) and liver (**d**) of mice infected with 3 × 10^4^ PFU of MHV-A59. Data represent mean ± SEM; n = 6–7 mice/group. * *p* < 0.05; unpaired Student’s *t*-test. ns: non-significant; L.O.D: limit of detection. (**e**–**h**) Hematological analysis (white blood cell count [WBC] (**e**), granulocyte (**f**) and lymphocyte (**g**) percentages, and granulocyte-to-lymphocyte ratio (**h**)) in the peripheral blood of mice infected with 3 × 10^4^ PFU of MHV-A59. Data represent mean ± SEM; n = 5–9 mice/group. * *p* < 0.05; # *p* < 0.05 when compared to their respective mock group. One-way ANOVA test.

Our group has recently shown that the instillation of a high-dose inoculum (3 × 10^5^ PFU) of MHV-A59 to mice through the intranasal route resulted in fatal infection, while a low-dose inoculum (3 × 10^4^ PFU) resulted in a mild, self-resolving disease [3]. To explore the role of Smurf1 in host resistance and regulation of inflammation in response to Betacoronavirus infection, we first inoculated via the intranasal route 6-to-8-week-old wild-type or *Smurf1*^−/−^ mice with 3 × 10^4^ (low-dose inoculum) or 3 × 10^5^ (high-dose inoculum) PFU of MHV-A59 and determined their survival and weight loss. We observed that 45% of wild-type mice inoculated with the high-dose inoculum of MHV-A59 succumbed between day 6 and 7 post-infection, while all *Smurf1*^−/−^ mice succumbed between day 2 and 6 post-infection (Figure 2a). Even though both wild-type and *Smurf1*^−/−^ mice inoculated with the high-dose inoculum underwent significant weight loss compared to their respective mock groups, the average body weight loss in *Smurf1*^−/−^ mice was significantly higher compared to wild-type mice (Figure 2b). When infected with a 10-fold lower dose inoculum of MHV-A59, wild-type and *Smurf1*^−/−^ mice did not succumb to infection (Figure 2a). However, both groups experienced significant weight loss compared to their respective mock groups (Figure 2b). Of note, weight loss in groups of mice infected with a low-dose was not as prominent as the one detected after infection with the high-dose of inoculum. To confirm whether intranasal delivery of a low-dose inoculum of MHV-A59 results in infection of target organs and systemic viral dissemination as previously shown [3], we assessed viral load in the lungs and liver of infected mice at 2, 5, and 11 days post-infection. We observed similar viral load recovery from the lungs of both wild-type and *Smurf1*^−/−^ at day 2 post-infection. However, on days 5 and 11 post-infection, virus titers in the lungs of wild-type mice were no longer recovered, while infective viral particles were still present in the lungs of *Smurf1*^−/−^ mice on day 5, suggesting a delayed clearance capacity (Figure 2c). We observed a similar viral load in the liver of both wild-type and *Smurf1*^−/−^ at day 5 post-infection, with absence of detection on days 2 and 11 post-infection (Figure 2d). These data suggest that intranasal infection with a low-dose inoculum of MHV-A59 results in a mild disease, with the infection initially establishing in the lungs before becoming systemic and ultimately infecting the liver.

It has been shown that infection of mice with MHV leads to leukopenia and lymphopenia [3,4,5]. Moreover, lymphopenia and neutrophilia have been associated with severity and mortality in patients with COVID-19 [27]. Given that infection with a low-dose inoculum of MHV-A59 leads to systemic infection and weight loss in both wild-type and *Smurf1*^−/−^ mice, we conducted hematological analysis at 2, 5, and 11 days post-infection to determine whether a low-dose inoculum stimulates changes in lymphocyte and granulocyte counts in the blood that could resemble systemic infection with Betacoronavirus. We observed significant leukopenia in *Smurf1*^−/−^ mice at day 2 post-infection, compared to their mock group, while wild-type mice did not present significant changes in leukocyte count in the blood at any period of the infection (Figure 2e). Both wild-type and *Smurf1*^−/−^ mice showed a significant increase in granulocytes (Figure 2f) and a decrease in lymphocytes (Figure 2g) on day 2 post-infection compared to their mock groups. However, while granulocyte and lymphocyte count returned to basal levels on days 5 and 11 post-infection in wild-type mice, the percentage of these cells in the blood of *Smurf1*^−/−^ mice remained abnormal until day 5, returning to normal on day 11 post-infection. It has been reported that the granulocyte-to-lymphocyte ratio (G/L) is a predictor of poor cancer prognosis, systemic inflammation, and a marker of severity in COVID-19 [28,29,30,31,32]. The analysis of G/L in infected mice showed a significant increase in both wild-type and *Smurf1*^−/−^ mice on day 2 post-infection compared to their respective mock groups (Figure 2h). Similar to the data of granulocyte and lymphocyte count, G/L returned to basal levels in wild-type mice at days 5 and 11 post-infection, while *Smurf1*^−/−^ mice still presented significantly high G/L at day 5 post-infection compared to their mock group or wild-type mice at the same period. These data suggest that intranasal infection with MHV-A59 results in hematological phenotypes that resembles a systemic inflammation. However, while hematological parameters returned to basal levels in wild-type mice after the second day of infection, *Smurf1*^−/−^ mice showed a delay in restoring those parameters. Overall, our findings suggest that Smurf1 is essential for host resistance against Betacoronavirus infection and modulates the systemic inflammatory response triggered by the infection.

### 3.3. Smurf1 Differentially Modulates Inflammatory Responses in the Lungs and Liver and Ameliorates Liver Damage in Mice Infected with Betacoronavirus

**Figure 3:**
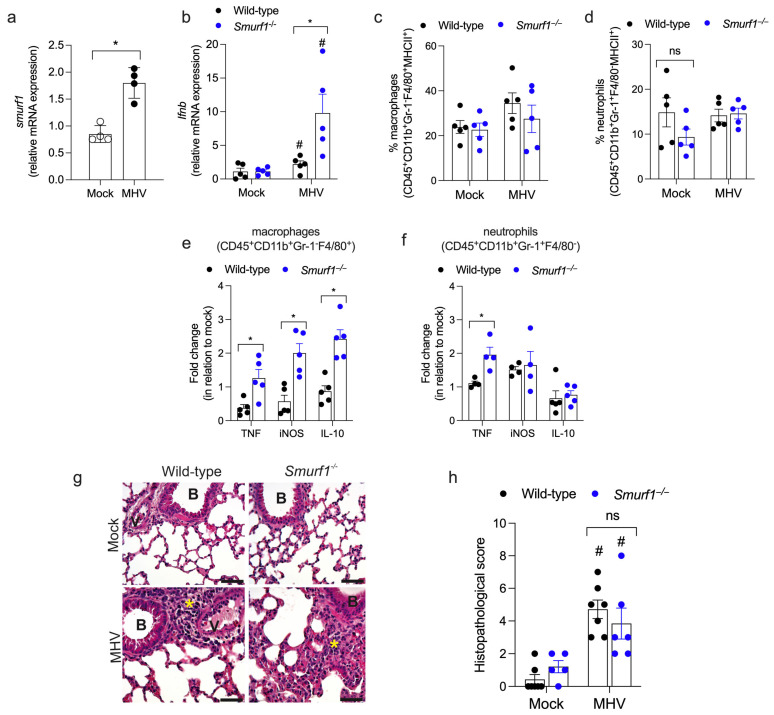
Smurf1 regulates the expression of mRNA for IFN-β and the inflammatory profile of macrophages and neutrophils in the lungs of infected mice. (**a**) Expression of Smurf1 mRNA in the lungs of wild-type mice infected with a low-dose of MHV-A59 at day 5 post-infection. Data represent mean ± SEM; n = 4 mice/group. * *p* < 0.05; unpaired Student’s *t*-test. (**b**) Expression of IFN-β mRNA in the lungs of infected mice. Data represent mean ± SEM; n = 4–5 mice/group. * *p* < 0.05; # *p* < 0.05 when compared to their respective mock group; unpaired Student’s *t*-test. (**c**,**d**) Flow cytometry analysis of activated macrophages (CD45^+^CD11b^+^Gr-1^-^F4/80^+^MHCII^+^) (**c**) and neutrophils (CD45^+^CD11b^+^Gr-1^+^F4/80^-^MHCII^+^) (**d**) in lungs from infected mice. Data represent mean ± SEM; n = 5 mice/group. ns: non-significant. (**e**,**f**) Analysis of macrophages (**e**) and neutrophils (**f**) positive for TNF, iNOS, or IL-10 in the lungs of infected mice. Data represent mean ± SEM; n = 4–5 mice/group. * *p* < 0.05; unpaired Student’s *t*-test. (**g**) Hematoxylin and eosin staining of lung sections from infected mice. Asterisk: mononuclear/polymorphonuclear cells. B: bronchiole; V: venules: Bars, 100 mm. (**h**) Histopathological score. Data represent mean ± SEM; n = 5–7 mice/group. # *p* < 0.05 when compared to their respective mock group; ns: non-significant; two-way ANOVA test.

Since *Smurf1*^−/−^ mice infected with a low-dose inoculum of MHV-A59 showed abnormal hematological parameters at day 5 post-infection, indicating potential inflammatory imbalance, we performed an additional analysis on lung and liver samples collected at day 5 post-infection. We observed that Smurf1 mRNA is upregulated in the lungs of wild-type mice upon infection with MHV-A59 (Figure 3a). To elucidate the role of Smurf1 in lung inflammation, we quantified TNF, IL-6, CXCL1, CCL5, and CCL2 production by ELISA. No significant changes in these cytokine levels were observed in either wild-type or *Smurf1*^−/−^ lungs upon MHV-A59 infection compared to their respective mock controls (Supplementary Figure S1). However, MHV-A59 infection induced IFN-β mRNA expression in both genotypes, with *Smurf1*^−/−^ mice exhibiting a significantly higher upregulation compared to wild-type mice (Figure 3b). A flow cytometry analysis revealed a similar percentage of activated macrophages and neutrophils (positive for MHC class II) in the lungs of infected mice (Figure 3c,d). To characterize the inflammatory profile of these cells, we quantified the proportion of macrophages and neutrophils positive for TNF, iNOS, or IL-10. Specifically, we calculated the fold increase in the percentage of marker-positive cells in infected animals (Supplementary Figure S2) relative to the percentage of positive cells in mock controls of the same genotype, referred to as fold change in relation to mock. We then compared this fold change between the wild-type and *Smurf1*^−/−^ groups. Our data showed that MHV-A59 infection significantly increased the proportions of TNF^+^, iNOS^+^, and IL-10^+^ macrophages, as well as TNF^+^ neutrophils, in the lungs of *Smurf1*^−/−^ animals compared to wild-type animals (Figure 3e,f). The histopathological analysis of lung parenchyma showed that wild-type and *Smurf1*^−/−^ mice exhibited similar alterations upon MHV-A59 infection. Notably, infected mice displayed lungs with marked perivascular and peribronchiolar mononuclear/polymorphonuclear leukocyte infiltrates (indicated by asterisks), alongside points of cellular death characterized by pycnotic nuclei. Additionally, some specimens exhibited small regions of interstitial, alveolar, and/or vascular edema (Figure 3g). Overall, the pulmonary histopathological injury was classified as moderate (Figure 3h).

**Figure 4:**
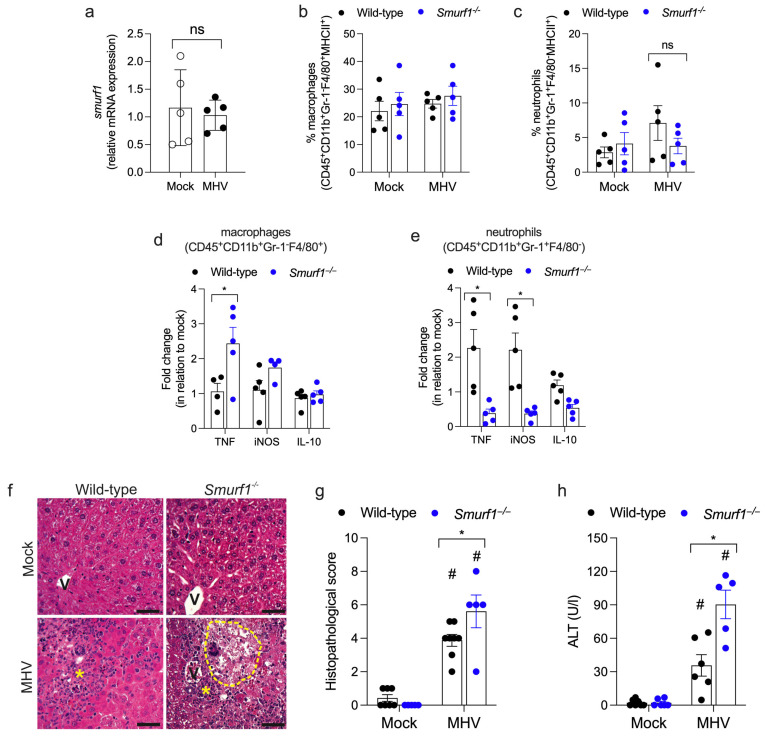
Smurf1 modulates the inflammatory profile of macrophages and neutrophils in the liver and ameliorates liver damage in mice infected with Betacoronavirus. (**a**) Expression of Smurf1 mRNA in the liver of wild-type mice infected with a low-dose of MHV-A59 at day 5 post-infection. Data represent mean ± SEM; n = 5 mice/group; unpaired Student’s *t*-test. ns: non-significant. (**b**) and (**c**) Flow cytometry analysis of activated macrophages (CD45^+^CD11b^+^Gr-1^-^F4/80^+^MHCII^+^) (**b**) and neutrophils (CD45^+^CD11b^+^Gr-1^+^F4/80^-^MHCII^+^) (**c**) in liver from infected mice. Data represent mean ± SEM; n = 5 mice/group. ns: non-significant. (**d**,**e**) Analysis of macrophages (**d**) and neutrophils (**e**) positive for TNF, iNOS or IL-10 in liver from infected mice. Data represent mean ± SEM; n = 4–5 mice/group. * *p* < 0.05; unpaired Student’s *t*-test. (**f**) Hematoxylin and eosin staining of liver sections from infected mice. Asterisk: leukocyte accumulation; dashed line: necrosis area; V: venules: Bars, 100 mm. (**g**) Histopathological score. (**h**) Serum alanine transaminase (ALT) levels from infected mice. Data represent mean ± SEM; n = 5–7 mice/group.* *p* < 0.05; # *p* < 0.05 when compared to their respective mock group; two-way ANOVA test.

In contrast to the lungs, MHV-A59 infection did not induce the expression of mRNA for Smurf1 in the liver of wild-type mice (Figure 4a). In addition, the analysis of mRNA for IFN-β and TNF revealed that MHV-A59 infection did not induce the expression of these mediators in the liver of either wild-type or *Smurf1*^−/−^ mice (Supplementary Figure S3a,b). However, both genotypes showed increased levels of IL-6 mRNA following MHV-A59 infection compared to the mock groups (Supplementary Figure S3c). No significant differences in IL-6 expression were observed between the infected wild-type and *Smurf1*^−/−^ mice. Similar to the lungs, livers displayed a comparable percentage of activated macrophages and neutrophils (positive for MHC class II) at day 5 post-infection (Figure 4b,c). Notably, the liver from *Smurf1*^−/−^ mice exhibited a higher ratio of macrophages TNF^+^ compared to the wild-type mice (Figure 4d and Figure S4). Conversely, the liver from *Smurf1*^−/−^ mice showed a decreased ratio of neutrophils positive for TNF and iNOS (Figure 4e and Figure S4). The histopathological analysis revealed increased cellularity within the sinusoidal capillaries in wild-type mice, indicative of inflammatory infiltrate, along with mild vasodilation and localized leukocyte accumulation (Figure 4f, asterisks). The *Smurf1*^−/−^ mice displayed these features with more frequent cell death and significant necrosis (Figure 4f, dashed line). MHV-A59 infection induced hepatic injury in both genotypes, but it was more accentuated in the *Smurf1*^−/−^ mice, as evidenced by the histopathological score (Figure 4g) and elevated serum alanine transaminase (ALT) levels (Figure 4h). Our results suggest that Smurf1 may not play a role in the recruitment of macrophages and neutrophils to the lungs and liver during Betacoronavirus infection, but it is essential in regulating the inflammatory profile of these cells that infiltrate the organs. Specifically, in the lungs, Smurf1 negatively modulates IFN-β mRNA in parenchyma and downregulates inflammatory factors by macrophages and neutrophils. In contrast, in the liver, Smurf1 does not affect IFN-β mRNA expression, and it upregulates TNF and iNOS by neutrophils while downregulating TNF by macrophages. Collectively, these findings imply that Smurf1 plays a key role in controlling lung and liver inflammatory responses during Betacoronavirus infection.

## 4. Discussion

Betacoronaviruses cause a spectrum of human illnesses, ranging from the common cold to the severe COVID-19. Understanding how host factors work to regulate the immune response to these viruses is crucial for the development of effective vaccines and therapeutic strategies. In this article, we showed that the host ubiquitin ligase Smurf1 is a critical factor in fine-tuning inflammatory immune responses and resistance against Betacoronavirus infection. Using a murine model of infection with MHV-A59, we found that mice lacking Smurf1 were more susceptible to intranasal coronavirus inoculation. *Smurf1*^−/−^ mice displayed hematological changes related to systemic inflammation; increased IFN-β mRNA in lungs; and higher expression of inflammatory markers, such as TNF and iNOS, by pulmonary macrophages and neutrophils, which was associated with delayed viral clearance in this organ. Moreover, we observed elevated levels of hepatic injury characterized by heightened cell death and necrosis, an increased ratio of TNF-positive macrophages within the livers, and heightened serum levels of ALT.

It has been reported that a significant factor contributing to the high mortality rates in COVID-19 patients is the uncontrolled inflammation triggered by lung macrophages and neutrophils, which leads to severe tissue damage and organ dysfunction [10,25]. Similarly, studies on experimental murine Betacoronavirus infection have shown that lung and liver immunopathology are correlated with dysregulated inflammation characterized by infiltration of macrophages and neutrophils, secretion of high levels of inflammatory mediators, and cell death [3,5,6,7,9]. The high mortality and the poor prognosis of COVID-19 has also been associated with the production of TNF during inflammatory responses to the infection. Our group has shown that genetic or pharmacological deletion of TNF receptor 1 (TNFR1) during experimental MHV-3 intranasal infection prevents vascular, respiratory, systemic changes, and death, as well as osteoporotic changes resulting from infection [5,33,34]. These findings emphasize the critical role of innate immune responses mediated by macrophages, neutrophils, and inflammatory mediators such as TNF in the immunopathology of Betacoronavirus infection. Here, we demonstrated that macrophages derived from *Smurf1*^−/−^ mice secreted increased levels of the proinflammatory mediators TNF and *CXCL1 in vitro*, and the lungs from *Smurf1*^−/−^ mice presented higher ratios of macrophages and neutrophils positive for TNF and iNOS upon infection with MHV-A59. In addition to the lungs, the liver from *Smurf1*^−/−^ mice presented an increased ratio of macrophages producing TNF and accentuated histopathological tissue damage, accompanied by increased serum levels of ALT, indicating disrupted inflammatory response in the absence of Smurf1. Based on our findings, we hypothesize that Smurf1 may regulate macrophage inflammation in Betacoronavirus infection by targeting for ubiquitination and degradation intracellular substrates involved in signaling cascades triggered upon virus recognition. This is supported by previous findings that Smurf1 directly interacts with and degrades molecules related to antiviral innate responses such as MAVS [17]; STAT1 [18]; TRAF3; TRAF6 [19]; and USP25, a deubiquitination enzyme that interacts with TRAF3 and TRAF6 [20]. Dysregulated degradation of these substrates in macrophages in the absence of Smurf1 may lead to the overproduction of pro-inflammatory factors with subsequent alterations in lung physiology and liver damage. The imbalance of inflammation is additionally demonstrated by the increased expression of IFN-β mRNA in the lungs of *Smurf1*^−/−^-infected mice, which could be due to heightened signaling of pathways related to virus recognition, thus providing further evidence that supports our hypothesis that Smurf1 may function as a regulatory component in intracellular signaling pathways involved in viral recognition by macrophages. Despite the fact that we observed an increased number of TNF^+^ macrophages in both the lungs and liver of *Smurf1*^−/−^ mice, significant tissue damage was only detected in the liver. We believe this discrepancy can be explained by two main factors: I) intrinsic characteristics of the virus, and II) the intrinsic roles of Smurf1 in liver physiology. Firstly, because MHV-A59 initially replicates in the respiratory tract and subsequently disseminates systemically and it is depurated in the liver [2], its presence in the lungs is transient, which could explain the absence of significant lung damage in *Smurf1*^−/−^, compared to wild-type mice. We hypothesize that the ultimate presence of MHV-A59 in liver may result in more pronounced and sustained systemic inflammatory alterations in *Smurf1*^−/−^ mice, contributing to the increased liver damage. These alterations are illustrated by changes in hematological parameters and leukopenia, which may enhance the susceptibility of *Smurf1*^−/−^ animals to infection, despite the similar lung injury observed. Second, Smurf1 may play intrinsic roles in liver physiology, potentially regulating processes such as cell death and metabolism, or other mechanisms that remain to be identified. Although no significant histopathological damage was observed in the lungs of *Smurf1*^−/−^ mice following low-dose inoculation, we suggest that the increased mortality rates in these mice during high-dose infection may be attributed to heightened macrophage- and neutrophil-mediated inflammatory responses and lung dysfunction, as well as inflammatory liver damage. Together, these data suggest that Smurf1 may represent a promising pharmacological target for the treatment and prevention of hyperinflammation caused by Betacoronavirus infection.

Betacoronaviruses undergo a complex intracellular replication cycle that consists of several stages, including cell attachment and entry, replication, viral particle assembly, and cell egress [35]. It has been shown that the activity of ubiquitin ligases may be involved in the cell egression phase of some RNA viruses. For example, the Ebola Virus VP40 protein was shown to interact with the WW domain of host WWP1 ubiquitin ligase to facilitate its egression and diffusion [36]. Our in vitro data showed reduced viral titers in the supernatants from the *Smurf1*^−/−^ macrophages infected with MHV-A59. Given that Smurf1 has two WW domains in its structure [37], we speculate that in infected macrophages, Smurf1 may interact with a yet-to-be-identified MHV-A59 substrate through its WW domains, potentially mediating virus egression. It is possible that Smurf1 interacts with the MHV-A59 spike protein in a manner similar to its recently demonstrated physical interaction with the SARS-CoV-2 spike protein [21] to promote virus egression. In addition to mediating viral cell egression, ubiquitin ligases are involved in cell autonomous mechanisms related to the degradation of viral proteins and viral clearance. It was recently shown that the E3 ubiquitin ligase RNF5 targets the SARS-CoV-2 envelope (E) protein for degradation by the ubiquitin-proteasome system, and RNF5-mediated degradation of E inhibits SARS-CoV-2 replication [38]. Smurf1 has been reported to interact with and mediate the targeting of Sindbis virus and herpes simplex virus capsids to autophagosomes in HeLa and murine embryonic fibroblast cells through virophagy [39], a cellular process that targets viral components for degradation within lysosomes [40]. Our in vivo data show that at day 2 post-infection, both wild-type and *Smurf1*^−/−^ mice exhibited similar viral titers in the lungs. However, by day 5, the virus was detectable only in the lungs of *Smurf1*^−/−^ mice, which suggest that MHV-A59 is cleared from the lungs of wild-type mice after 48 h post-infection possibly through mechanisms mediated by Smurf1. In this context, we propose that in vivo Smurf1 may play an additional role in promoting virus clearance by mediating degradative intracellular autonomous mechanisms such as virophagy.

Overall, the data presented in this study support a model in which Smurf1 acts as a multifunctional molecule that plays diverse roles during Betacoronavirus infection in vivo. By modulating macrophages inflammatory responses, Smurf1 is essential to fine-tunning inflammation triggered by viral recognition in innate immune cells and preventing tissue damage. Additionally, Smurf1 may mediate intracellular mechanisms that promote viral clearance in infected organs, highlighting its critical role in regulating immune response to viral pathogens.

## Data Availability

The original contributions presented in the study are included in the article/Supplementary Materials, further inquiries can be directed to the corresponding author.

## Supplementary Materials

The following supporting information can be downloaded at: https://www.mdpi.com/article/10.3390/pathogens13100871/s1, Figure S1: Production of inflammatory mediators in the lungs of infected mice; Figure S2: Flow cytometry analysis of macrophages and neutrophils in lungs from infected mice; Figure S3: Expression of mRNA for IFN-β, TNF and IL-6 in the liver of infected mice; Figure S4: Flow cytometry analysis of macrophages and neutrophils in liver from infected mice.
