# Supplementary figures and images for: E3 Ubiquitin Ligase Smurf1 Regulates the Inflammatory Response in Macrophages and Attenuates Hepatic Damage during Betacoronavirus Infection
Source: Pathogens. 2024 Oct 3;13(10):871. doi: 10.3390/pathogens13100871 (PMC11510589; doi:10.3390/pathogens13100871)

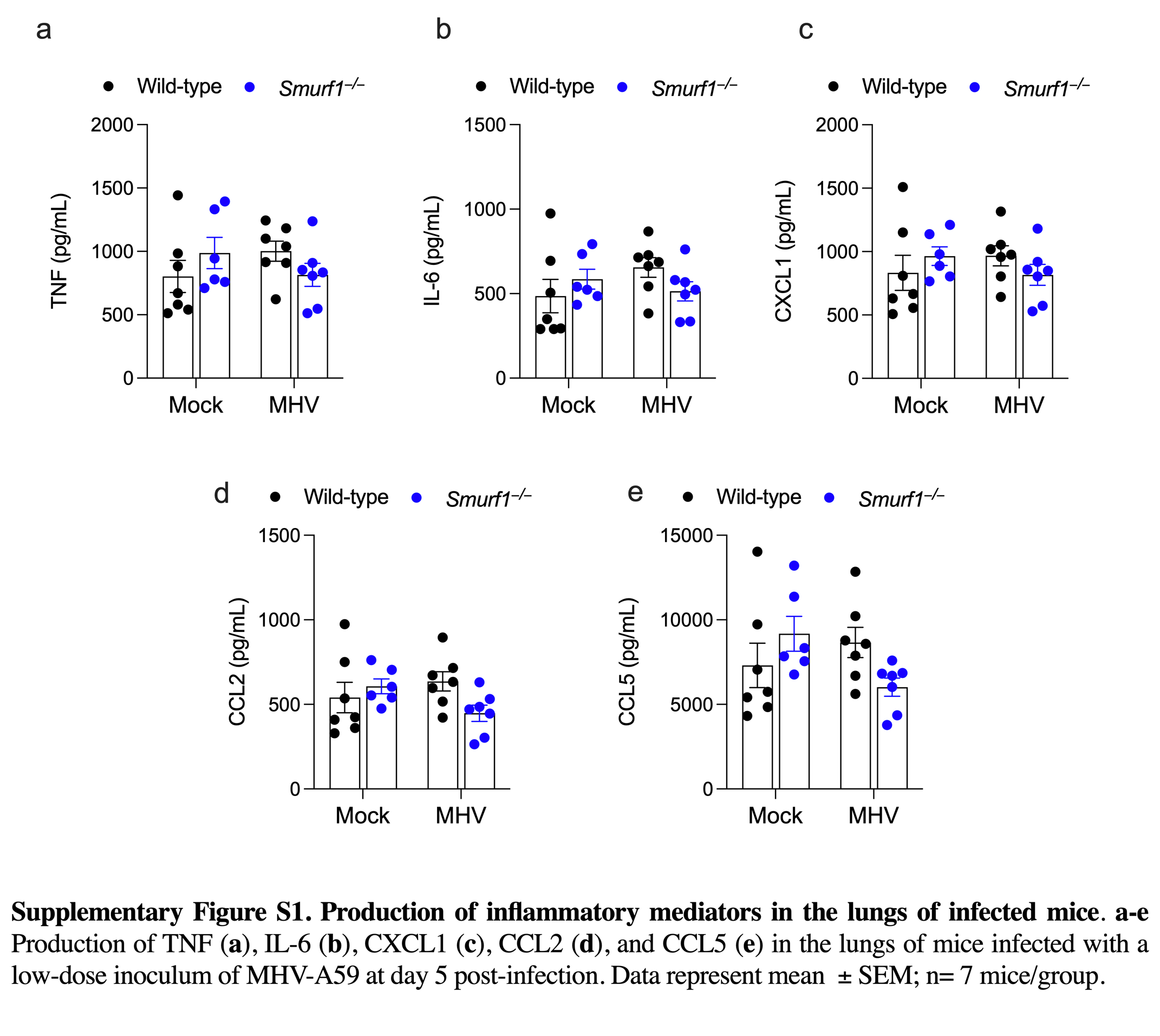

Supplement: Supplementary file 1 [file pathogens-13-00871-s001.zip › Supplementary Fig S1.tiff]

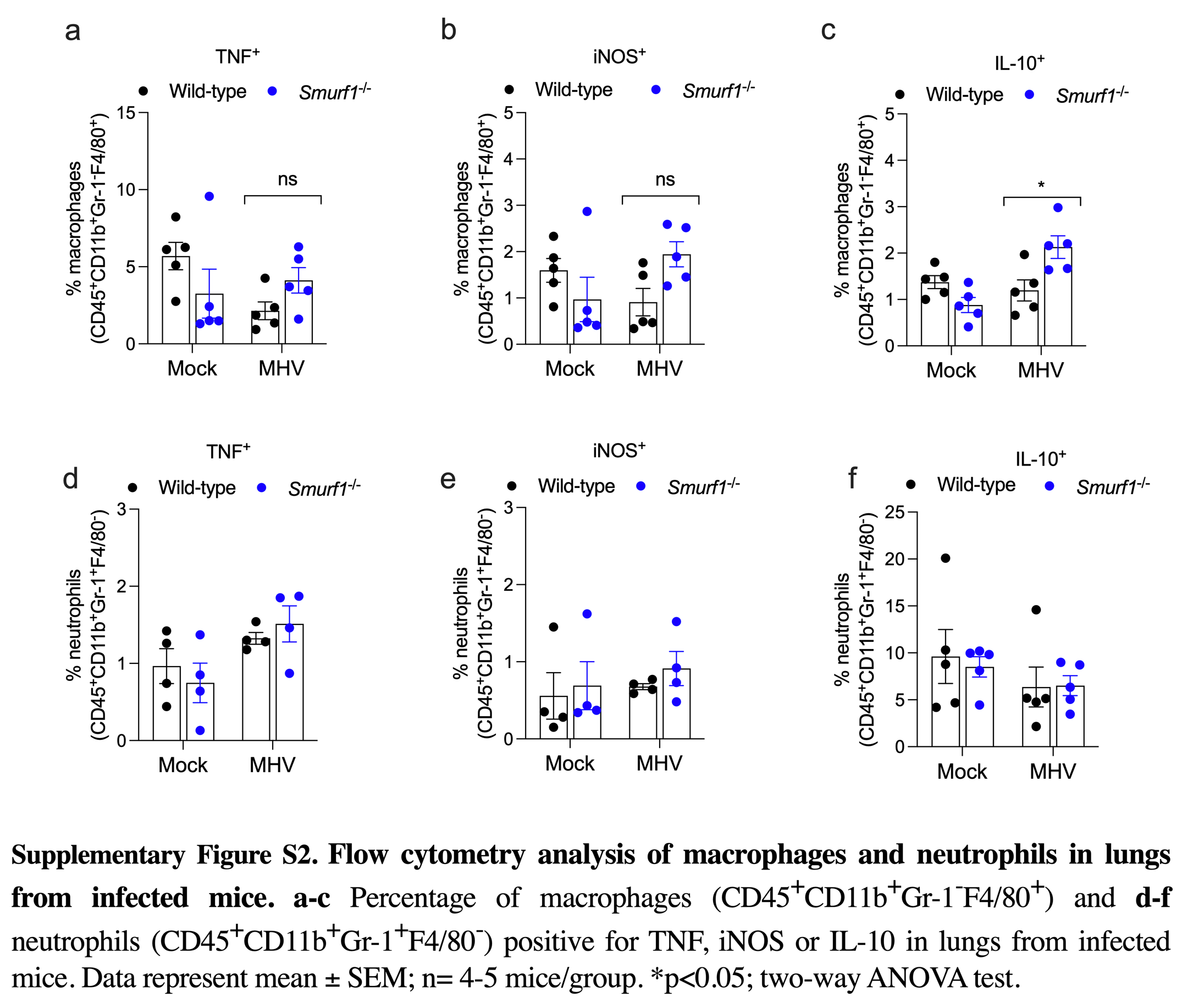

Supplement: Supplementary file 1 [file pathogens-13-00871-s001.zip › Supplementary Fig S2.tiff]

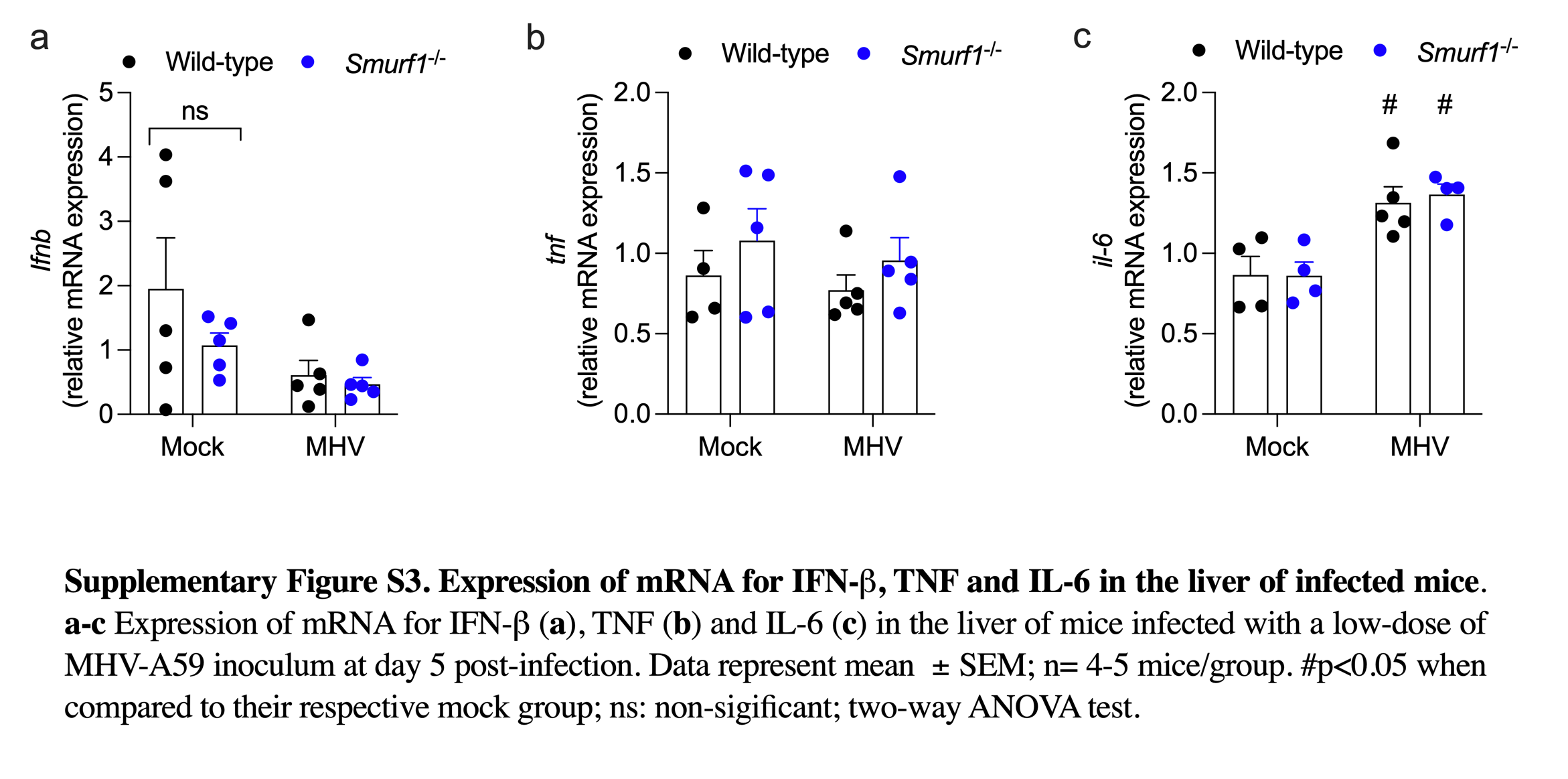

Supplement: Supplementary file 1 [file pathogens-13-00871-s001.zip › Supplementary Fig S3.tiff]

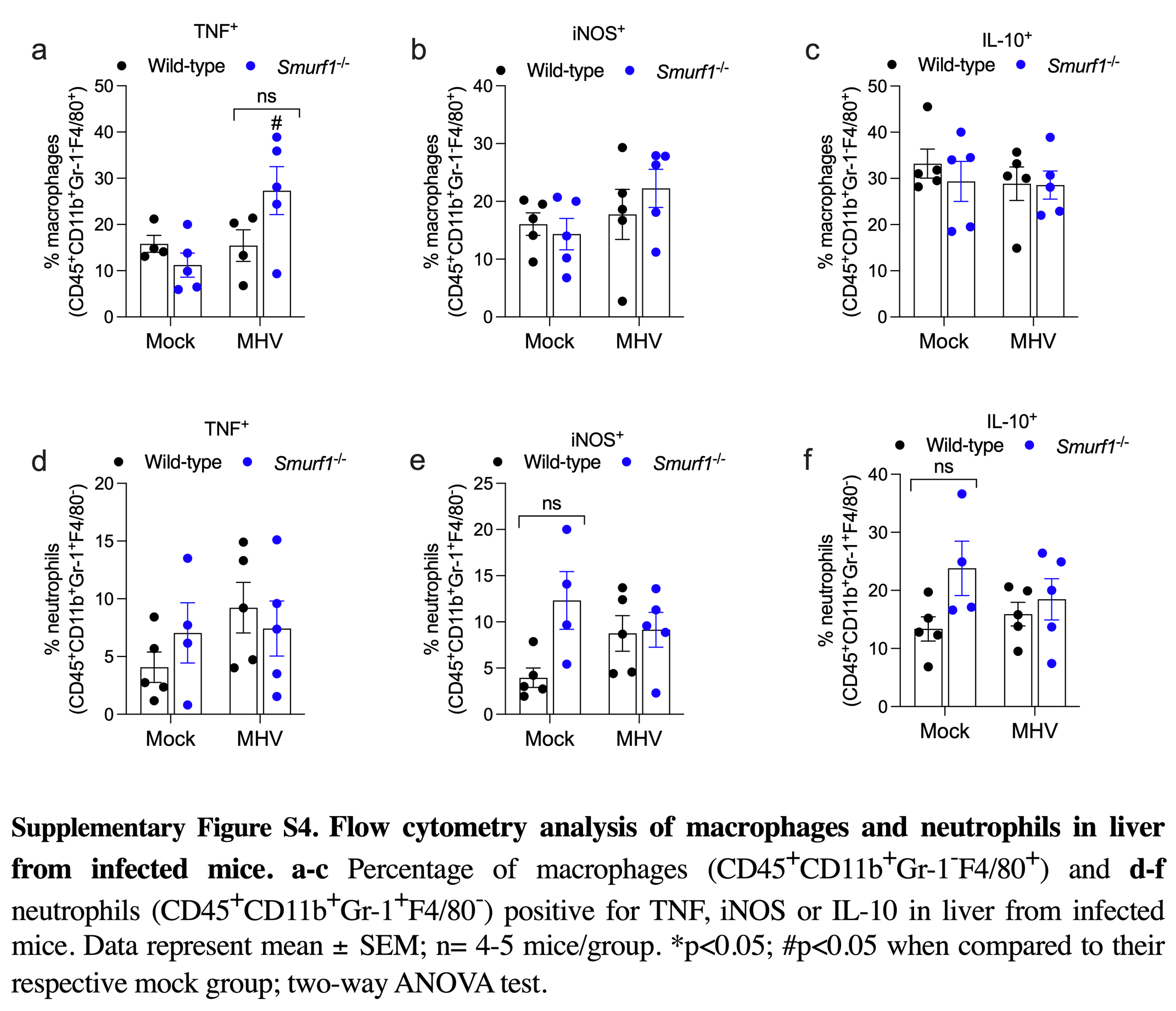

Supplement: Supplementary file 1 [file pathogens-13-00871-s001.zip › Supplementary Fig S4.tiff]
